# Multi-Objective Optimization Method for High-Efficiency and Low-Consumption Wire Rope Greasing Process

**DOI:** 10.3390/s25072053

**Published:** 2025-03-25

**Authors:** Fan Zhou, Yuemin Wang, Ruqing Gong, Binghui Tang

**Affiliations:** 1 College of Power Engineering, Naval University of Engineering, Wuhan 430030, China; zf422725587@163.com (F.Z.); aoi97s@163.com (R.G.); tangbinghui0816@163.com (B.T.); 2 College of Intelligent Manufacturing, Wuhan Polytechnic, Wuhan 430079, China

**Keywords:** wire rope greasing, objective function, constraints, optimization parameters

## Abstract

Wire rope greasing is essential for protecting wire ropes from corrosion and wear. To address issues such as low maintenance efficiency and excessive grease usage, this study proposes a high-efficiency, low-consumption optimization control method for the wire rope greasing process. A time objective function for the greasing process and a consumption objective function for grease are established. Considering the actual constraints of greasing equipment performance and greasing quality, a multi-objective optimization model is developed with greasing speed, greasing thickness, grease flow rate, and greasing time as the optimization parameters. The model aims to achieve high efficiency (minimizing greasing process time) and low consumption (minimizing grease consumption). Weight coefficients are introduced to transform the multi-objective optimization model into a single-objective optimization model, which is then solved using an improved genetic algorithm. The effectiveness of the model is validated through a specific case study, and a sensitivity analysis of the weight coefficients of the objective functions in the optimization model is conducted. This research provides valuable support for wire rope greasing process planning and improvement.

## 1. Introduction

Wire ropes are widely used in various engineering fields requiring high strength and wear resistance, such as aerospace, lifting and transportation, marine engineering. In complex environments, wire ropes are subjected to significant tensile and bending stresses, as well as corrosion and wear [1,2,3,4]. To ensure normal operation and extend the service life of wire ropes, wire rope greasing is a crucial step. Greasing can effectively protect the wire rope surface from corrosion and wear, enhancing its service life and safety. However, the existing greasing control methods have issues such as uneven greasing and excessive or insufficient grease application, leading to negative impacts including reduced wire rope lifespan, increased maintenance costs, environmental pollution, and health hazards to operators. Therefore, it is essential to develop an optimized control method for the wire rope greasing process.

To reveal the effects of different lubricants and lubrication conditions on the friction behavior of wire ropes, scholars have conducted in-depth research on wire rope friction and wear. Dyson, C.J. et al. demonstrated that tightly braided wire ropes (6 × 19) have smaller inter-wire gaps, hindering uniform lubricant penetration, while loosely braided wire ropes (6 × 37) allow easier penetration and distribution. At cross-contact points, uneven lubricant distribution may intensify local wear [5]. Huang et al. studied the friction and wear behavior of wires under different lubrication conditions and corrosion levels [6]. Peng Y et al. further explored the friction and wear issues of multiple wires in wire ropes, finding that the coefficient of friction (COF) decreases with increasing transverse load and tension, affecting the performance and safety of wire ropes [7]. Liu Dabiao’s research showed that friction significantly enhances the stiffness of wire ropes with layered spiral structures and causes periodic changes in contact forces [8]. Krishan emphasized the importance of fretting behavior on wire rope performance, highlighting the need to study key variables to improve reliability and service life [9]. Zhang Y et al. studied the application of lanthanum stearate-modified lubricating oil in mine hoist wire ropes, demonstrating that optimizing the viscosity and consistency of lubricants can significantly improve tribological performance, thereby enhancing lubrication efficiency and reducing wear [10]. These studies provide a scientific basis for optimizing greasing methods and selecting appropriate grease thickness.

Optimizing greasing parameters and reducing grease consumption without compromising efficiency are crucial for improving the overall greasing efficiency, cost effectiveness, and environmental impact. Wang W. et al. designed a device for cleaning and lubricating wire ropes along the twist direction of the strands, determining lubrication parameters through dynamic modeling analysis, addressing issues such as old grease accumulation, and meeting the maintenance needs of cableway wire ropes [11]. Halim Mgs et al. optimized and developed a specialized tool that reduced wire rope lubrication time by 67%, while improving efficiency and reducing environmental impact and harm [12]. Xia Yifan et al. conducted parametric modeling and finite element analysis of triangular-strand wire ropes using ABAQUS/CAE 2023 software, verifying the model’s accuracy through experiments [13]. Wu Zhang used the Frenet–Serret theory and Pro/ENGINEER Wildfire 5.0 software to perform spatial geometric modeling of 6 × 7 + IWS wire ropes, achieving the precise modeling of endless wire ropes [14]. Fang G. et al. designed a six-wheel wire rope climbing robot for the online cleaning and safety inspection of sluice wire ropes, providing an effective tool for sluice maintenance [15]. These studies have made good progress in accurately analyzing grease consumption and determining greasing process parameters through wire rope structure analysis and maintenance device design, but less attention has been paid to determining optimal grease consumption based on greasing parameters.

To address the efficiency and environmental burden challenges in wire rope greasing, this study focuses on optimizing two objectives: greasing process time and grease consumption. The greasing parameters considered include greasing speed, greasing thickness, grease flow rate, and greasing time, aiming to reduce greasing process time and grease consumption by optimizing these parameters. Various traditional and non-traditional optimization techniques are available for such problems. One method is the genetic algorithm (GA), which simulates natural evolution processes to optimize multi-dimensional non-linear problems and is a current trend in oiling process optimization [16]. Many researchers have reported using the GA to solve multi-objective optimization problems. For example, Chen Zhou et al. proposed an improved NSGA-II algorithm for optimizing hydraulic interconnected suspension systems, significantly improving ride comfort and roll stability [17]. Zhao Shangnan et al. presented an enhanced NSGA-II algorithm for imaging optical systems to achieve multi-objective optimization [18]. Shang Yunyue et al. combined response surface methodology with the GA to optimize the structure of a ball mill barrel, successfully reducing the barrel mass by 15.624% while maintaining structural strength [19]. However, the traditional GA tends to converge prematurely and become stuck in local optima. Considering this, this study explores an improved GA combining adaptive penalty functions and crossover operations for optimization calculations and simulations. This allows each generation to retain some infeasible solutions, enabling global optimization on both the feasible and infeasible solution sides.

Motivated by the foregoing discussion, a method for greasing process parameter optimization with a high efficiency and low consumption is proposed. This method combines greasing process parameters with greasing process time and grease consumption to solve the optimization problem. Firstly, a multi-objective optimization model is established with greasing process time and grease consumption as the objective functions. Secondly, weight coefficients are introduced to transform the multi-objective optimization model into a single-objective optimization model. Thirdly, the single-objective optimization model and the multi-optimization model will each be solved with an improved GA. Lastly, an illustrative example is given to demonstrate the effectiveness of the multi-objective optimization model.

## 2. Establishment of the Greasing Process Parameter Optimization Model

### 2.1. Determination of Optimization Variables

In the greasing process, greasing speed (*v*), greasing thickness (*d*), grease flow rate (*L*), and greasing time (*t*_1_) are key variables affecting the greasing effectiveness and performance. To comprehensively consider these factors, a greasing process parameter optimization variable model can be established to achieve the best greasing effect in actual production. In this model, the optimization variable vector *X* can be expressed as follows:

$$X={\left(v,d,L,t_{1}\right)}^{T}$$

### 2.2. Determination of Optimization Objectives

This paper mainly optimizes the greasing process from two aspects: high efficiency (greasing process time) and low consumption (grease consumption). The optimization objectives are greasing process time and grease consumption.

#### 2.2.1. Greasing Process Time Objective Function

The complete greasing process time comprises the greasing time, the greaser replacement time, and the auxiliary process time. The mathematical model of the objective function for the greasing process time can be expressed as follows [20]:

(1) $$T_{p}=t_{1}+t_{2}\frac{t_{1}}{T}+t_{0}$$

(2) $$t_{1}=\frac{l_{w}}{v}$$

(3) $$T=\frac{K_{T}G_{T}}{p^{\frac{1}{m}}v^{\frac{1}{n}}h^{\frac{1}{u}}}$$

where $T_{p}$ is the greasing process time; $l_{w}$ is the greasing length; v is the greasing speed; $t_{0}$ is the auxiliary process time; $t_{1}$ is the greasing time; $t_{2}$ is the greaser replacement time; T is the greaser service life; $K_{T}$ is the correction coefficient; $G_{T}$ is the friction coefficient; p is the contact pressure; h is the allowable wear thickness; and *m*, *n*, *u* are wear-related coefficients. Substituting (2) and (3) into (1), the greasing process time objective function becomes the following:

(4) $$T_{p}=t_{1}+\frac{t_{1}t_{2}p^{\frac{1}{m}}v^{\frac{1}{n}}h^{\frac{1}{u}}}{K_{T}G_{T}}+t_{0}$$

#### 2.2.2. Grease Consumption Objective Function

**Figure 1:**
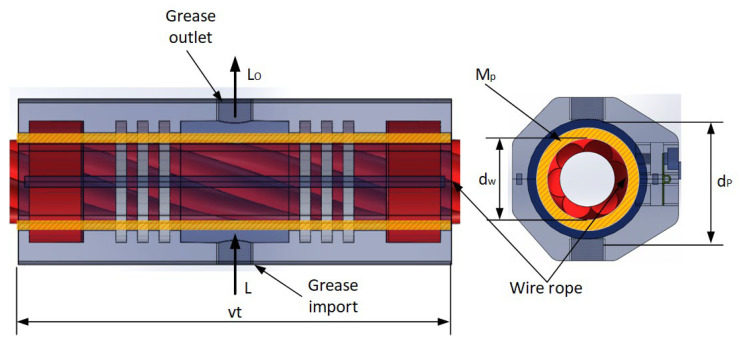
Schematic diagram of grease consumption calculation.

The grease consumption in the greasing process primarily includes two components: the grease consumed for coating the surface of the wire rope and the additional grease consumption due to load conditions (under load conditions, the greasing pressure on the wire rope increases, causing the grease to be squeezed out of the contact area, thereby resulting in additional grease consumption), as shown in Figure 1. Thus, the volume grease consumption $M_{p}$ is expressed as below [11]:

(5) $$M_{p}=Lt_{1}=M_{o}+M_{c}=\frac{\pi }{4}\frac{\left(D_{p}^{2}-D_{w}^{2}\right)}{1000}vt_{1}+L_{O}t_{1}$$

(6) $$d=\frac{D_{p}-D_{w}}{2}$$

where $M_{p}$ is the grease consumption, *t*_1_ is the greasing time, $M_{o}$ is the grease consumed for coating the surface of the wire rope, $M_{c}$ is the additional grease consumption due to load conditions, *d* is the greasing thickness, *D_p_* is the diameter after greasing, *D_w_* is the diameter of the wire rope, *L* is the grease inlet flow, *L_O_* is the grease outlet flow, and the formula for calculating the grease consumption is as follows:

(7) $$M_{p}=[\frac{\pi d\left(D_{w}+d\right)v}{1000}+L_{O}]t_{1}$$

### 2.3. Optimization Model

The multi-objective parameter optimization problem aiming to minimize grease consumption and greasing process time is defined as Equation (8). Moreover, g(X)≤0 represents the operational constraints given in Equation (9):

(8) $$\left\{\begin{array}{c} minf\left(X\right)={\left(minT_{p},{minM}_{p}\right)}^{T}\\ X={\left(v,d,L,t_{1}\right)}^{T}\\ s.t.g(X)\leq 0 \end{array}\right.$$

(9) $$\left\{\begin{array}{c} \frac{\pi Dn_{min}}{1000}\leq v\leq \frac{\pi Dn_{max}}{1000}\\ d_{min}\leq d\leq d_{max}\\ L_{min}\leq L\leq L_{max}\\ S_{min}\leq vt_{1}\leq S_{max}\\ \frac{F_{c}v}{1000\varphi }\leq P_{max} \end{array}\right.$$

where $n_{min}$ and $n_{max}$ are the minimum and maximum motor speeds, *D* is the drive wheel diameter, $d_{min}$ and $d_{max}$ are the minimum and maximum allowable greasing thicknesses, $L_{min}$ and $L_{max}$ are the minimum and maximum allowable flow rates, $S_{min}$ and $S_{max}$ are the minimum and maximum greasing distances, $F_{c}$ is the traction force, $P_{max}$ is the maximum power of the greasing device, and *φ* is the power efficiency coefficient.

In the above mathematical expressions, Equation (8) sets the optimization objective to simultaneously minimize greasing process time and grease consumption. Subsequently, Equation (9) provides the operational constraints, typically setting bounds for the greasing process parameters (greasing speed, greasing thickness, grease flow rate, and greasing time).

## 3. Model Solution Based on Improved Genetic Algorithm

### 3.1. Multi-Objective Function Transformation

For multi-objective optimization problems, it is often difficult to achieve optimality for all objectives simultaneously, and finding Pareto solutions for multi-objective optimization problems is challenging. A common method for solving multi-objective optimization problems is to reduce the number of objectives, transforming the multi-objective optimization problem into a single-objective optimization problem [21,22,23]. In the process of transforming multi-objective into single-objective optimization, the weighted sum method is frequently used. This paper uses the weighted sum method to transform the above multi-objective optimization problem into a single-objective optimization problem. The corresponding single-objective optimization function is as follows:

(10) $$minf\left(X\right)=min(w_{1}T_{p}+w_{2}M_{p})$$

where $w_{i}$ is the weight coefficient, and $w_{1}+w_{2}=1$. For determining the weights, methods such as the Analytic Hierarchy Process (AHP), fuzzy evaluation, expert scoring, and group decision-making can be used [24,25,26]. Since the greasing process time objective function $T_{p}$ and the grease consumption objective function $M_{p}$ have different dimensions, the sum operation cannot be carried out. Dimensionless processing is required, as follows:

(11) $$T_{p}^{*}=\frac{T_{p}\left(v,d,L,t_{1}\right)-T_{min}}{T_{max}-T_{min}}$$

(12) $$M_{p}^{*}=\frac{M_{p}\left(v,d,L,t_{1}\right)-M_{min}}{M_{max}-M_{min}}$$

where $T_{min}$ and $T_{max}$ are the minimum and maximum values of the greasing process time objective function, $M_{min}$ and $M_{max}$ are the minimum and maximum values of the grease consumption objective function. Substituting (11) and (12) into (10), the dimensionless single-objective optimization function is as follows:

$$\begin{array}{c} minf\left(v,d,L,t_{1}\right)=min\left(w_{1}T_{p}^{*}+w_{2}M_{p}^{*}\right)\\ =min\left(w_{1}\frac{T_{p}\left(v,d,L,t_{1}\right)-T_{min}}{T_{max}-T_{min}}+w_{2}\frac{M_{p}\left(v,d,L,t_{1}\right)-M_{min}}{M_{max}-M_{min}}\right) \end{array}$$

### 3.2. Improved Genetic Algorithm for Greasing Process Optimization

Since the greasing process optimization model is a problem containing linear and non-linear constraints, a commonly used method is the penalty function method, which transforms constrained optimization problems into unconstrained problems. However, the penalty function method has drawbacks such as complex calculations and low accuracy. The genetic algorithm (GA) is an adaptive method that has been widely used to solve complex search and optimization problems, but the traditional genetic algorithm is prone to premature and local convergence. Therefore, this paper proposes an improved algorithm that incorporates penalty functions into the fitness evaluation, constructing a fitness function with adaptive penalty factors that change with the solution feasibility and adaptive crossover rates. This allows each generation to retain some infeasible solutions, enabling global optimization on both the feasible and infeasible solution sides. The specific process is detailed below.

#### 3.2.1. Population Size and Encoding Selection

The initial population is randomly selected in the variable optimization space, and the population size should be set as a reasonable integer *N*, whose value plays an important role in algorithm convergence. If *N* is too small, the optimization performance is poor, and the algorithm is prone to local optima. If *N* is too large, the computational complexity and workload increase sharply, affecting evolutionary efficiency. According to the literature [27], the population size *N* generally ranges from 20 to 100. Binary encoding is used for each variable, forming a string, i.e., the chromosome, which is the operation object of the GA. An appropriate string length *m* is selected under precision requirements to reduce the GA computational load.

#### 3.2.2. Fitness Function with Adaptive Penalty Term

In the GA, the fitness size determines the quality of individuals and whether they can enter the next generation. To prevent a few highly fit individuals from rapidly reproducing in the population, causing premature convergence, the fitness function must be set reasonably to improve overall algorithm performance. This paper uses greasing process time and grease consumption as objective functions, solving for their minimum values by introducing the previously analyzed constraints as penalty functions into the optimization objective, constructing the GA fitness function as follows:

(13) $$F\left(x\right)=f\left(x\right)+p\left(x\right)$$

(14) $$p\left(x\right)=\left\{\begin{array}{cc} 0; & When\;x\;is\;feasible\\ r\left(t\right)\sum_{i=1}^{m}g_{i}\left(x\right); & \;lb_{i}\leq g_{i}\left(x\right)\leq ub_{i}\\ r\left(t\right)\sum_{i=1}^{m}{\left(g_{i}\left(x\right)-lb_{i}\right)}^{2}; & \;g_{i}\left(x\right)<lb_{i}\\ r\left(t\right)\sum_{i=1}^{m}{\left(g_{i}\left(x\right)-ub_{i}\right)}^{2}; & g_{i}\left(x\right)>ub_{i} \end{array}\right.$$

where *x* is the chromosome; F(x) is the fitness function; f(x) is the objective function; p(x) is the penalty function; $\;ub_{i}$, $lb_{i}$ are the upper and lower bounds of the *i*-th unsatisfied constraint. In Equation (14), the traditional penalty factor *r* is usually set as a constant. When *r* is large, the algorithm converges quickly in the early search stages but loses optimal solutions in later stages due to low precision. When *r* is small, the search is prone to local optima, hindering a global search. Therefore, in order to set r(t) reasonably and improve algorithm performance, this paper constructs an adaptive penalty factor r(t) that differs from traditional penalty factors and depends on the evolution generation *t*, with the relationship determined by the following:

(15) $$r\left(t+1\right)=\left\{\begin{array}{c} \alpha \times r\left(t\right),\;\alpha <1\\ r\left(t\right)\\ \beta \times r\left(t\right),\;\beta >1 \end{array}\right.$$

Equation (15) shows that, if the best individuals in the previous *t* generations are all feasible, α<1 is set to reduce the penalty factor; if the best individuals in the previous *t* generations are all infeasible, β>1 is set to increase the penalty factor; if there are both feasible and infeasible best individuals, the penalty factor remains unchanged from the previous generation. Using adaptive penalty functions allows the penalty factor to change with the solution feasibility, retaining both the feasible and some infeasible solutions, effectively avoiding premature convergence or local optima and obtaining global optima.

#### 3.2.3. Genetic Algorithm Operations

(1) Selection: Based on individual fitness evaluation, the purpose of selection is to directly pass optimized individuals to the next generation or generate new individuals through crossover and pass them to the next generation. For a population size *N* and individual fitness $f_{i}$, the individual selection probability can be calculated as follows:

(16) $$p_{i}=f_{i}/\sum_{k=1}^{N}f_{N}$$

(2) Adaptive crossover rate: The crossover operator randomly selects η(2≤η≤24) parent individuals from the population, recombines them to form *η* offspring individuals, and inserts them into the new population. Typically, *η* is set to 2. The crossover probability $P_{c}\left(t\right)$ determines the number of offspring individuals. To improve search capability, the crossover rate is set to adaptively change with genetic iterations, enhancing the algorithm’s global optimality. Its expression can be written as follows:

(17) $$P_{c}\left(t+1\right)=\left\{\begin{array}{c} P_{C}\left(t\right)\times \frac{\left(f_{max}-f\right)}{\left(f_{max}-f_{avg}\right)},\;f\geq f_{avg}\\ P_{C}\left(t\right),\;f<f_{avg} \end{array}\right.$$

where $f_{max}$ is the maximum fitness in the population, $f_{avg}$ is the average fitness of the population, *f* is the larger fitness of the two parents, $P_{c}\left(t\right)$ is the *t*-th generation of the crossover rate. For individuals with a fitness higher than the population average, $P_{c}\left(t\right)$ is reduced from the previous generation, allowing individuals with excellent genes to enter the next generation smoothly. For individuals with a fitness lower than the population average, the crossover rate remains unchanged from the previous generation.

(3) Mutation rate: Each individual in the population is scanned, and genes are mutated with a mutation probability $P_{m}$. In the GA, mutation effectively prevents premature convergence. Therefore, the mutation probability $P_{m}$ should not be too large, generally ranging from 0.0001 to 0.1000.

#### 3.2.4. Algorithm Steps

**Figure 2:**
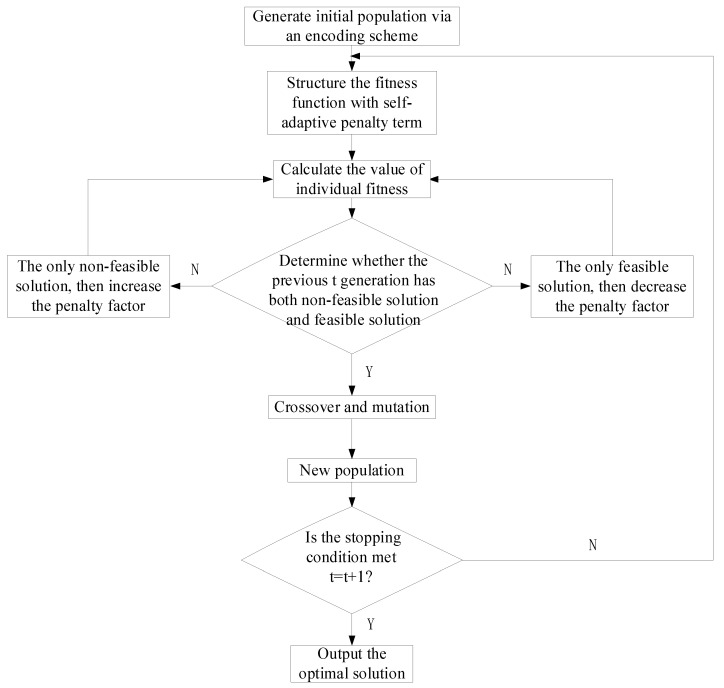
Flowchart of the improved genetic algorithm.

The steps of the improved GA proposed in this paper are as follows: (1) problem description and abstraction; (2) population initialization, determining variables and their bounds, randomly generating the parent population, and setting basic algorithm parameters; (3) constructing the fitness function with adaptive penalty terms, setting the initial penalty factor $r\left(1\right)=1$, and calculating individual fitness; (4) judging the feasibility of solutions in the previous *t* generations (if there are both feasible and infeasible solutions, the penalty factor remains unchanged; if all the solutions are feasible, set $\alpha =\frac{1}{2}$ to reduce the penalty factor; if all the solutions are infeasible, set β=2 to increase the penalty factor); (5) genetic operations: selection, setting crossover rate, and mutation rate; (6) checking whether the stopping conditions are met (if not, set t≔t+1 and go to step (2) until (7) output the optimal solution, as shown in Figure 2.

## 4. Case Study

### 4.1. Experimental Conditions

**Figure 3:**
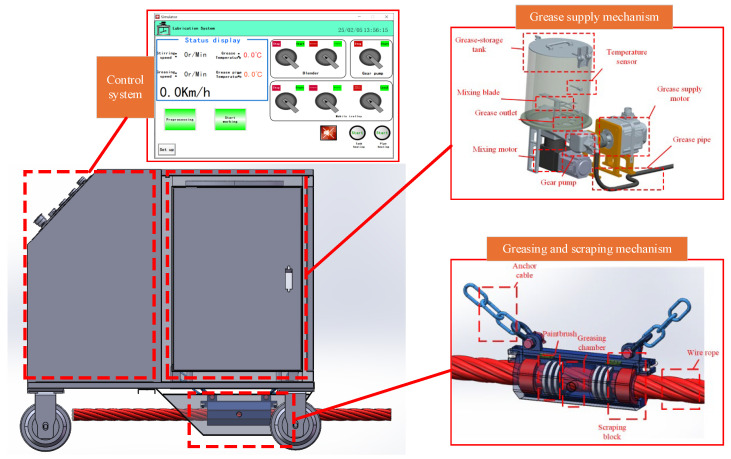
Greasing device structure diagram.

Experimental device: The research team developed a smart wire rope greasing device using a mobile cart as the chassis, which precisely controlled the cart’s movement speed through a control system. The cart platform was equipped with an execution mechanism and control system, including oiling and scraping mechanisms, a grease supply mechanism, limit switches, heating wires, and several sensors. By controlling the grease supply motor speed to drive the gear pump, the grease flow rate was adjusted. Grease flowed into the greasing and scraping mechanisms, and, as the cart moved, the greaser was pulled uniformly, causing the greasing chamber to move linearly along the wire rope, applying grease evenly to the entire wire rope. The greasing device structure diagram is shown in Figure 3.

Lubricant properties: The wire rope length was about 20 m; the wire rope diameter was about 36.5 mm, 6 × 37 construction. For tightly braided wire ropes, we selected high-viscosity and high-consistency grease to enhance its adhesion and anti-extrusion performance. The grease used in this study was in a solid state at room temperature and exhibited strong adhesion under normal conditions. Due to the poor fluidity of high-adhesion grease, it is necessary to preheat the grease using the heating mechanism in the greasing device before applying it. The specific parameters of the grease are shown in Table 1.

Experimental requirements: The greasing time was $t_{1}\leq 1$ min: the greasing thickness was *d* = 0.5 mm; the greasing coverage needed to reach 100%; and the grease layer needed to uniformly cover the wire rope. The specific greasing device parameters are shown in Table 2, and the calculated correlation coefficients are shown in Table 3 and Table 4.

### 4.2. Optimization Results

(1) The determined constraints were as follows:

$$s.t\left\{\begin{array}{c} 4.8\leq v\leq 60\\ 0\leq L\leq 10\\ t_{1}\leq 1\\ vt_{1}\geq 20\\ v\leq 48 \end{array}\right.$$

(2) The improved GA optimization parameters were as follows: initial population size *N* = 100; maximum generations 100; initial penalty factor $r\left(1\right)=1$; crossover rate $P_{c1}=0.95$; mutation rate $P_{m}=0.06$, δ=0.5, φ=2.

To verify the necessity of the proposed model, a set of experiments was conducted to study the relationship between the greasing process parameters, greasing process time, and grease consumption. Using Matlab R2023a, the optimization calculations were performed, yielding single-objective and multi-objective optimization results, as shown in Table 5.

**Figure 4:**
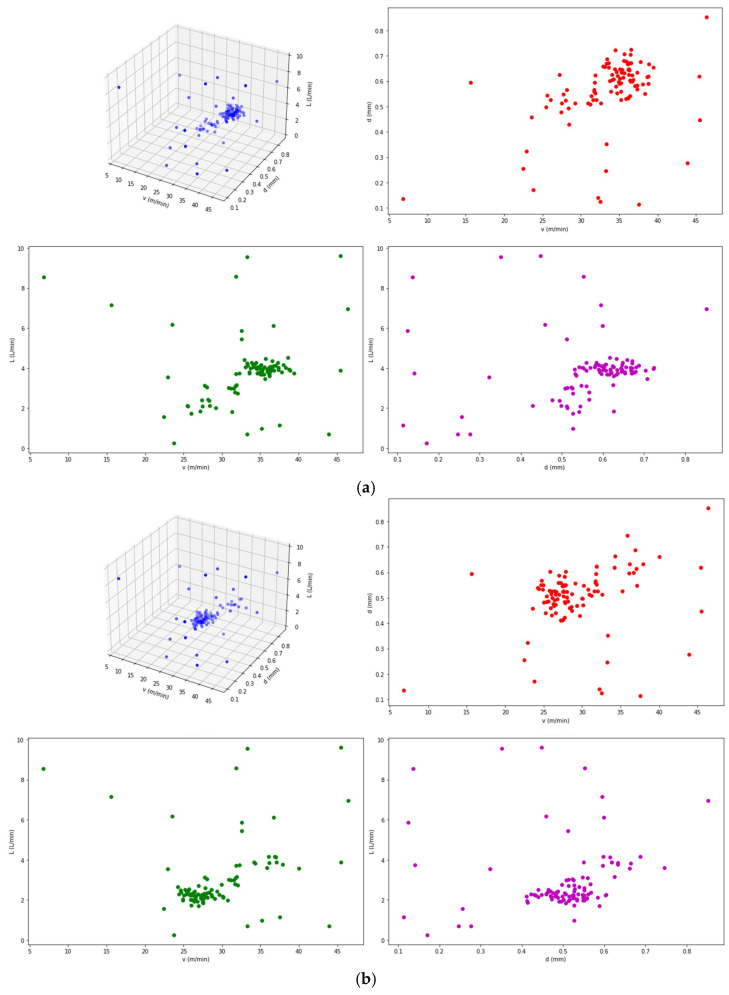

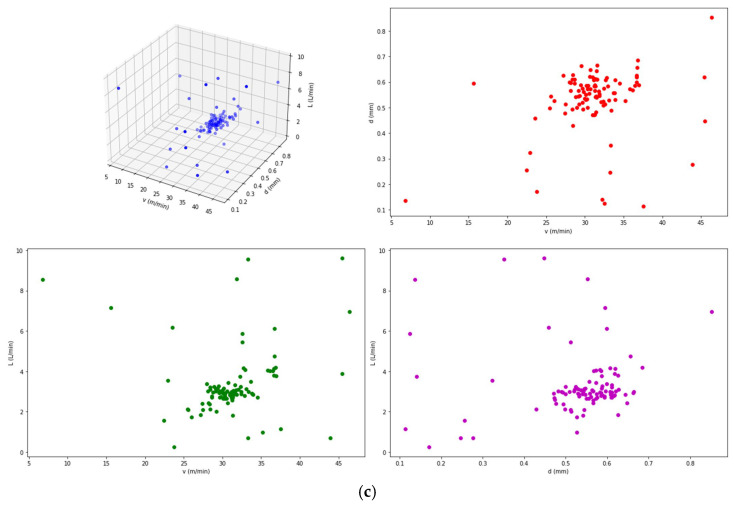
Results of single-objective optimization and multi-objective optimization; (**a**) single-objective optimization for greasing process time; (**b**) single-objective optimization for grease consumption; (**c**) multi-objective optimization.

(3) The optimization results were as follows: Scenario 3 (Figure 4c) was the optimal result of the optimization model considering both greasing process time and grease consumption objectives. Scenario 1 (Figure 4a) and 2 (Figure 4b) were the optimal results of single-objective optimization considering greasing process time and grease consumption, respectively. Scenario 1 exhibited the shortest greasing process time, followed by Scenario 3, while Scenario 2 had the longest greasing process time. This indicates that the greasing process time was optimally controlled in Scenario 1, whereas the increased process time in Scenario 2 was due to the consideration of optimization requirements for grease consumption. Scenario 2 demonstrated the lowest grease consumption, followed by Scenario 3, while Scenario 1 showed the highest grease consumption. This indicates that Scenario 2 performed best in terms of minimizing the grease consumption, whereas Scenario 1, which prioritized the optimization of the greasing process time, resulted in a relatively higher grease consumption. Scenario 3 achieved a certain balance between the greasing process time and the grease consumption.

### 4.3. Optimization Result Analysis

**Figure 5:**
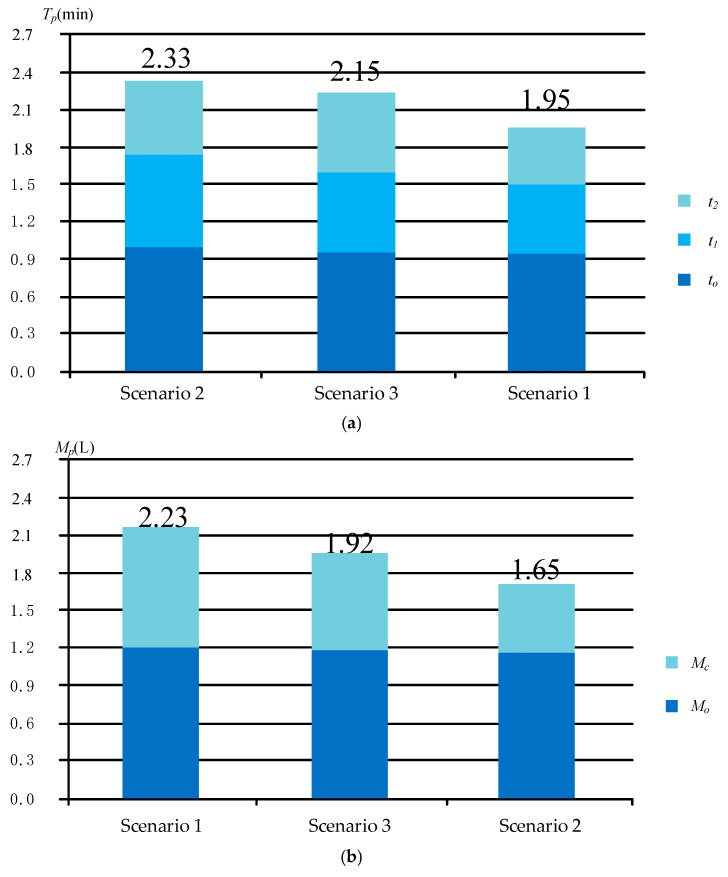
Multi-objective and single-objective optimization of greasing process time and grease consumption composition: (**a**) optimization for greasing process time; (**b**) optimization for grease consumption.

Scenario 1, which was the efficiency-oriented optimization, resulted in a higher greasing speed, larger grease flow rate, larger grease outlet flow, and more grease consumption than in other cases. The increase in *M_c_* was more significant, leading to an overall rise in the total grease consumption, as depicted in Figure 5b. Scenario 2, which was the consumption-oriented optimization, resulted in a lower greasing speed, smaller grease flow rate, smaller grease outlet flow, and longer greasing time than in the other cases. The increase in *t*_1_ was more significant, leading to an overall rise in the greasing process time, as depicted in Figure 5a. The multi-objective optimization balanced both objectives, achieving a 13.9% reduction in grease consumption and a 12.2% improvement in greasing efficiency compared to the single-objective optimization. The composition of the multi-objective and single-objective optimization of greasing process time and grease consumption is shown in Figure 5.

### 4.4. Experimental Implementation

**Figure 6:**
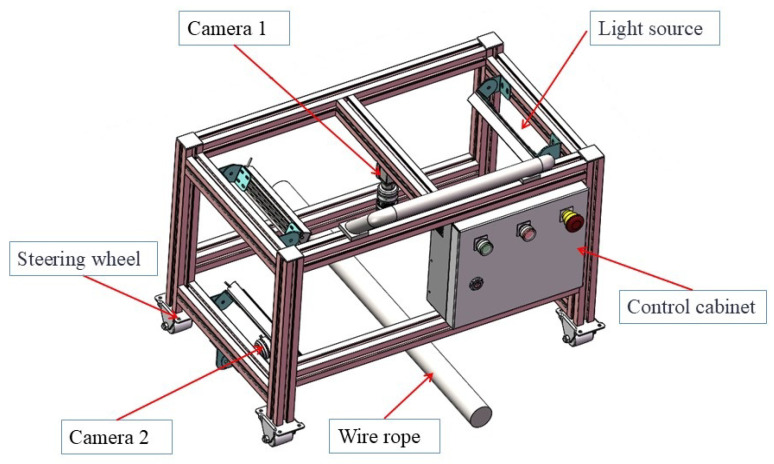
Wire rope greasing quality visual measurement system.

The greasing parameters obtained through the multi-objective optimization (Table 4, Scenario 3) were input into the greasing device to carry out the greasing operation. The quality of the wire rope greasing was monitored in real time using a visual measurement system that provided a non-invasive and precise method for evaluating the greasing thickness, as shown in Figure 6.

**Figure 7:**
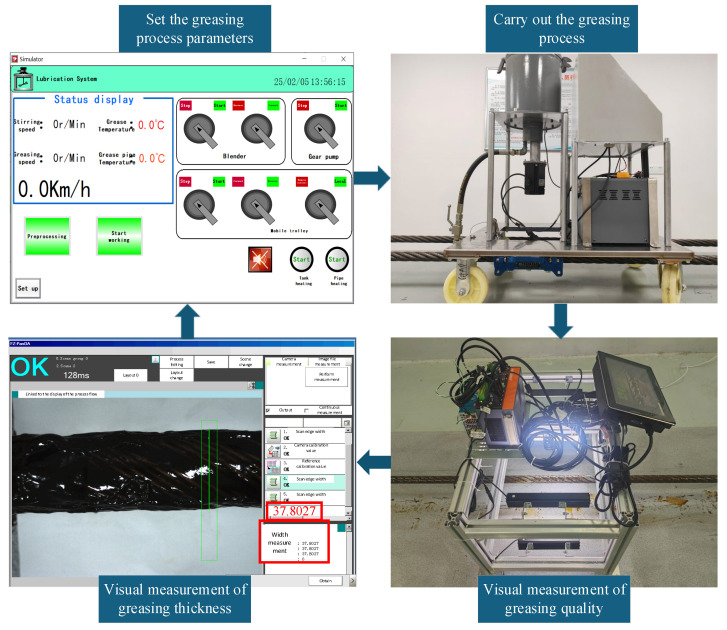
Greasing optimization implementation process and greasing effect.

The measured diameter of the wire rope before greasing was 36.65 mm, and the diameter after greasing was 37.80 mm, resulting in a greasing thickness of 0.575 mm. This measured thickness closely aligned with the optimized greasing thickness predicted by the multi-objective optimization model (the optimized thickness result for Table 4, Scenario 3 was 0.56 mm), demonstrating the accuracy of the optimization process. The visual measurement system used for quality control exhibited a precision of ±0.02 mm in thickness measurement, with a repeatability error of less than 1.5%. The specific greasing effect is illustrated in Figure 7, which visually confirms the uniform and consistent application of the lubricant.

## 5. Discussion

In the optimization analysis, equal weights were selected to balance the consideration of the two objectives, greasing process time and grease consumption; to avoid bias toward either objective; and to establish a baseline for comparison. To investigate the impact of the weight coefficients on the optimization results, a sensitivity analysis of the weight coefficients was conducted. As detailed in Table 6, the sensitivity analysis of the weight coefficients revealed a clear trade-off between the greasing process time and the grease consumption.

**Figure 8:**
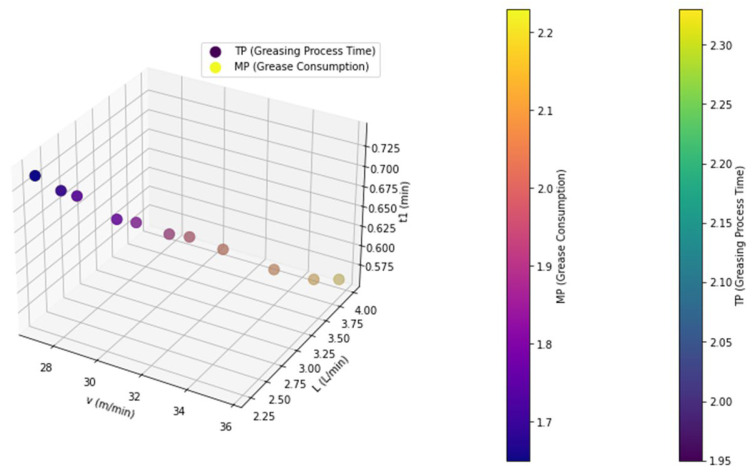
Sensitivity analysis of weight coefficients for greasing process time and grease consumption.

From Table 6, a clear trade-off between the greasing process time *T_P_* and the grease consumption *M_P_* can be observed. As the weight *w*_1_ (weight for greasing time) decreased from 1 to 0, *T_P_* gradually increased from 1.95 min to 2.33 min, while *M_P_* gradually decreased from 2.23 L to 1.65 L. This indicates that optimizing the greasing time leads to an increase in the grease consumption, while optimizing the grease consumption results in a longer greasing time. In practical applications, the selection of weights should be dynamically adjusted based on specific requirements. For example, in high-efficiency production lines, a higher *w*_1_ value can be prioritized to shorten the greasing time, thereby improving production efficiency; in scenarios in which grease costs are high, a higher *w*_2_ value can be chosen to reduce the grease consumption and lower costs, as shown in Figure 8. Through the multi-objective optimization method, an optimal balance between the greasing time and the grease consumption can be achieved, providing a scientific basis for industrial production.

## 6. Conclusions

(1) A multi-objective optimization model was established with the objectives of minimizing the greasing process time and the grease consumption, and optimizing parameters such as the greasing speed, greasing thickness, grease flow rate, and greasing time, aiming for high efficiency and low consumption.

(2) An improved genetic algorithm was employed to solve the optimization model. The effectiveness of the optimization model was verified through a specific case study, and a sensitivity analysis of the weight coefficients of the optimization objectives was conducted, laying a theoretical foundation for achieving high-efficiency and low-consumption greasing in enterprises.

(3) Due to the diversity of wire rope greasing processes, this study primarily focused on the optimization of a single-step greasing process for high efficiency and low consumption. However, in practice, most greasing processes involve multiple steps and procedures, and there may be multiple process routes to complete a greasing task. Therefore, the development of efficient and low-carbon optimization models for multi-step and multi-procedure processes, as well as the optimization of process routes, will be the focus of future research.

## Figures and Tables

**Table 1:** The specific parameters of the grease.

**Base Oil**	**Thickener**	**Dropping Point** **(°C)**	**Worked Penetration** **(0.1 mm)**	**Adhesion** **(L/min)**	**Preheating Temperature** **(°C)**
Synthetic hydrocarbon	Metal soap	78	355	High	50

**Table 2:** Specific greasing device parameters.

*n_min_* (r/min)	*n_max_* (r/min)	*L_min_* (L/min)	*L_max_* (L/min)	*P_max_* (kW)	*D* (mm)	*F_c_* (N)
10	125	0	10	1	152.4	750

**Table 3:** Calculated correlation coefficients.

*m*	*n*	*u*	*w*_1_	*w*_2_	*K_T_*	*Φ*
1/3	1/2	−1	0.5	0.5	0.6	0.6

**Table 4:** Calculated relevant parameters.

*L_w_* (m)	*D_W_* (mm)	*d* (mm)	*T*_0_ (min)	*T*_2_ (min)	*p* (Mpa)	*G_T_* (mm^3^/N.m)	*h* (mm)
20	36.5	0.5	1	0.5	2	10^3^	0.2

**Table 5:** Results of single-objective optimization and multi-objective optimization.

Scenario	1	2	3
*T_P_*	1.95	2.33	2.15
*M_P_*	2.23	1.65	1.92
*t*_1_	0.56	0.74	0.65
*v*	35.71	27.03	30.77
*L_w_*	20	20	20
*d*	0.59	0.51	0.56
*L*	3.98	2.23	2.95
*L*_0_	1.85	0.62	1.12

**Table 6:** Sensitivity analysis of weight coefficients.

Scenario Weight	1 (1,0)	2 (0.9,0.1)	3 (0.8,0.2)	4 (0.7,0.3)	5 (0.6,0.4)	6 (0.5,0.5)	7 (0.4,0.6)	8 (0.3,0.7)	9 (0.2,0.8)	10 (0.1,0.9)	11 (0,1)
*T_P_*	1.95	2.03	2.06	2.10	2.12	2.15	2.18	2.23	2.28	2.31	2.33
*M_P_*	2.23	2.15	2.08	2.03	1.98	1.92	1.85	1.78	1.74	1.69	1.65
*t*_1_	0.56	0.57	0.59	0.62	0.64	0.65	0.67	0.68	0.71	0.72	0.74
*v*	35.71	35.09	33.90	32.26	31.25	30.77	29.85	29.41	28.17	27.78	27.03
*L_w_*	20	20	20	20	20	20	20	20	20	20	20
*d*	0.59	0.59	0.58	0.57	0.56	0.56	0.55	0.55	0.53	0.51	0.51
*L*	3.98	3.77	3.53	3.28	3.10	2.95	2.76	2.62	2.45	2.35	2.23
*L*_0_	1.85	1.67	1.49	1.34	1.22	1.12	0.97	0.85	0.76	0.68	0.62

## Data Availability

Data are contained within the article.

## Nomenclature

*D*	Drive wheel diameter (mm)
*d*	Greasing thickness (mm)
*D_p_*	Diameter after greasing (mm)
*D_w_*	Diameter of wire rope (mm)
*d_min_*	Minimum allowable greasing thickness (mm)
*d_max_*	Maximum allowable greasing thickness (mm)
*F_c_*	Traction force (N)
*G_T_*	Friction coefficient
*h*	Allowable wear thickness (mm)
*K_T_*	Correction coefficient
*L*	Grease flow rate (L/min)
*L*_0_	Grease outlet flow (L)
*L_min_*	Minimum allowable flow rate (L/min)
*L_max_*	Maximum allowable flow rate (L/min)
*L_w_*	Greasing length (m)
*M_p_*	Grease consumption (L)
*M_o_*	Grease consumed for coating the surface of the wire rope (L)
*M_c_*	Additional grease consumption due to load conditions (min)
*m*, *n*, *u*	Wear-related coefficients
*n_min_*	Minimum motor speed (r/min)
*n_max_*	Maximum motor speed (r/min)
*P_max_*	Maximum power of the greasing device (Kw)
*p*	Contact pressure (Mpa)
*S_min_*	Minimum greasing distance (mm)
*S_max_*	Maximum greasing distance (mm)
*T*	Greaser service life (min)
*T_p_*	Greasing process time (min)
*t*_0_	Auxiliary process time (min)
*t*_1_	Greasing time (min)
*t*_2_	Greaser replacement time (min)
*v*	Greasing speed (m/min)
*w_i_*	Weight coefficient
*φ*	Power efficiency coefficient
